# Insights of different analytical approaches for estimation of budesonide as COVID-19 replication inhibitor in its novel combinations: green assessment with AGREE and GAPI approaches

**DOI:** 10.1186/s13065-023-00936-z

**Published:** 2023-03-14

**Authors:** Mohammed E. A. Hammouda, Amal A. El-Masry, Saadia M. El-Ashry, Dalia R. El-Wasseef

**Affiliations:** 1 Department of Medicinal Chemistry, Faculty of Pharmacy, Mansoura University, Mansoura, 35516 Egypt; 2 Department of Pharmaceutical Chemistry, Faculty of Pharmacy, Horus University - Egypt, New Damietta, Egypt; 3 Department of Pharmaceutical Chemistry, Faculty of Pharmacy, Delta University for Science and Technology, Gamasa, 35712 Egypt

**Keywords:** Azelastine, Budesonide, HPLC, Zero order, First derivative, Ratio first derivative, COVID-19, Inhalation corticosteroids, Allergic rhinitis, AGREE, GAPI

## Abstract

Simple, direct, rapid, and sensitive HPLC and spectrophotometric methods were established for simultaneous estimation of a novel combination of budesonide and azelastine (BUD/AZL) in their laboratory-prepared mixture and dosage form according to the medicinally recommended ratio 1:4.28. Budesonide is an important inhalation corticosteroid that plays a vital role in the inhibition of COVID-19 replication and cytokine production. The first chromatographic method was created for the simultaneous estimation of BUD epimers in the presence of AZL with excellent efficiency in a relatively short chromatographic run (< 9 min). The separation of BUD epimers with AZL was carried out on a C_18_ column using acetonitrile: phosphate buffer of pH 3.5 adjusted by 0.2 M orthophosphoric acid (40:60, v/v) as a mobile phase, UV detection at 230 nm and a flow rate of regulated at 2 mL/min. Besides, three spectrophotometric methods were applied for the simultaneous determination of the provided mixture adopting zero order, first order derivative, and ratio first derivative approaches. The Zero-order spectrophotometry was used for the determination of AZL in presence of BUD, where BUD shows no absorbance at 290 nm. The first derivative amplitude at 265 nm (^1^D_265_) (zero-crossing of AZL) and the ratio of first derivative amplitudes at 270 nm (^1^DD_270_) using 10.0 µg mL^−1^ AZL as divisor was chosen for the simultaneous determination of BUD in the presence of AZL in the binary mixture. The proposed methods were found to be rectilinear in the concentration range of (0.4–40.0 µg mL^−1^) and (0.05–40.0 µg mL^−1^) for BUD and AZL, respectively in the HPLC method. Whereas the concentration range for AZL in the zero-order method was (1.0–35.0 µg mL^−1^) and for BUD in the first derivative and ratio derivative method was (6.0–20.0 µg mL^−1^). Validation of the suggested approaches according to the ICH criteria was performed. Furthermore, to ensure the proposed approaches' greenness, The AGREE and GAPI metrics were utilized, and the afforded results revealed an excellent greenness of the proposed approaches.

**Supplementary Information:**

The online version contains supplementary material available at 10.1186/s13065-023-00936-z.

## Introduction

**Fig. 1:**
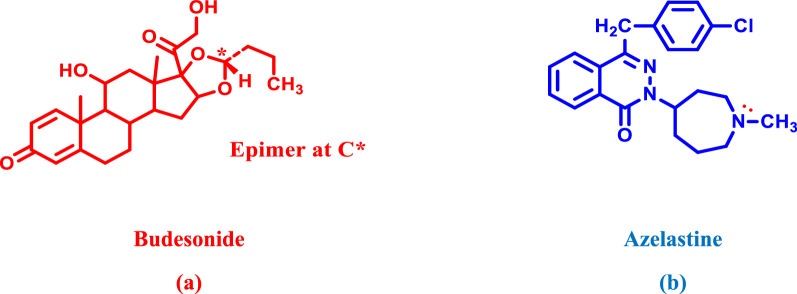
Chemical structures of **a** budesonide and **b** azelastine

Azelastine (AZL), chemically named as: (4-(4-chlorobenzyl)-2-[(4RS)-1-methylhexahydro-1*H*-azepin-4-yl]phthalazin-1(2*H*)-one hydrochloride [1]; Fig. 1b. It is a second generation relatively selective histamine H_1_ antagonist, which prevents mast cells implicated in the allergic reaction from releasing histamine and other mediators. AZL has been demonstrated for the inhibition of leukotrienes and platelet-activating factors. Furthermore, it inhibits the accumulation and degranulation of eosinophils at the site of allergic inflammation. It is recommended for the treatment of the symptoms of seasonal allergic rhinitis and conjunctivitis in adults and pediatric patients [1].

Budesonide (BUD), chemically named as (*R*,*S*)-16α,17α-butylidenedioxy-11β,21-dihydroxypregna-1,4-diene 3,20-dione [2]; Fig. 1a. is an anti-inflammatory, its affinity for the glucocorticoid receptor is approximately 200-fold greater than that of hydrocortisone and 15-fold greater than that of prednisolone. BUD is available in a combination of two epimers (22R and 22S) that resulted from adding an alkyl chain at a chiral C_22_ atom, where the ratio range of R:S epimers was within 60/40, 51/49 [1]. High glucocorticoid activity is observed in both epimers, with the 22R epimer having a twofold higher affinity than the 22S epimer [3].

The in vitro data suggests that treatment with the inhaled corticosteroid budesonide in conjunction with bronchodilators inhibits Human coronavirus 229E (HCoV-229E) multiplication and cytokine generation in bronchial epithelial cells, according to recent studies on the pandemic coronavirus [4]. Angiotensin-converting enzyme 2 (ACE2) gene expression is reduced in the sputum of Chronic obstructive pulmonary disease (COPD) and asthma patients who take inhaled corticosteroids (ICS) compared to those who do not [5, 6], suggesting that ICS may also inhibit SARS-CoV-2 penetration. Additionally, research in mice has demonstrated that ICS decreases ACE2 expression by preventing the production of type 1 Interferon (1 IFN) [5]. The resulting decrease in ACE2 expression may protect SARS-CoV-2 cellular entrance, even while inhibition of type 1 IFN production may impair host defense. These data suggest that the use of ICS especially budesonide in asthmatic and COPD patients may protect against Coronavirus disease 2019 (COVID-19) and be considered an interesting drug candidate for further development [7].

Upon reviewing the literature, it was discovered that several analytical techniques have been published for the determination of BUD in both pure form and pharmaceutical formulations, such as; spectrophotometry [8–12], high-performance liquid chromatography (HPLC) [13–20], liquid chromatography with tandem mass spectrometry (LC/MS/MS) [21], ultra-high performance liquid chromatography with tandem mass spectrometry (UPLC/MS/MS) [22, 23] and gas chromatography (GC) [24]. Moreover different analytical methods were established for the determination of AZL either in pure form or pharmaceutical preparations, such as; spectrophotometry [25–31], spectrofluorimetry [32], electrochemical methods [33–35], TLC [36, 37], HPLC [28, 38–41], high-performance thin layer chromatography (HPTLC) [42], LC/MS/MS [43, 44] and electrokinetic capillary chromatography (EKCC) [44].

A novel combination of (BUD and AZL) significantly reliefs allergic nasal symptoms (itchy nose and sneezing) as measured by the total nasal symptom score (TNSS) [45], where the results of scientific research showed that the use of BUD and AZL together led to significant improvements in nasal congestion that occurred more quickly than with either drug used alone in patients with seasonal allergic rhinitis [46].

The effectiveness and novelty of this combination encouraged the establishment of different analytical approaches utilized for the estimation of both drugs in their synthetic mixture and dosage forms. The innovation of the provided approaches is that there are neither spectrophotometric nor HPLC methods yet have been developed in the literature for their simultaneous estimation. Thus, it was imperative to develop new facile, sensitive, and effective methods for their determination. Different analytical parameters were studied and optimized, furthermore, the greenness of the proposed approaches was ensured by adopting the AGREE and GAPI metrics.

## Experimental

### Instruments

HPLC separation was performed with Knauer series P 6.1 L Chromatograph equipped with a 20 μL Rheodyne injector valve and a UV/VIS detector operated at 230 nm. Total Chrom Workstation (Massachusetts, USA) was utilized for the collection and processing of data. The mobile phase filtration was performed by 0.45 µm membrane filters (Millipore, Ireland). The pH was measured by A Consort P-901 pH meter.

A Lamb America Unico-USA series visible double-beam spectrophotometer (model S1200 equipped with 2 matched 1-cm path-length quartz cells) was adopted for the spectrophotometric analyses.

### Reagents and materials

BUD certified as 99.80% purity was kindly provided by Jayco Chemical Industries (Maharashtra, India), and its pharmaceutical preparation Rhinocort Aqua (Contains 32 µg/dose BUD, Batch no #170183) was obtained from the local pharmacy. Azelastine hydrochloride (AZL) certified as 99.80% purity was provided as a gift by European Egyptian Pharmaceuticals Industry, Alexandria, Egypt, and its pharmaceutical preparation Zalastin Metered Dose Inhaler (labeled to contain 1 mg mL^−1^ AZL, Batch no #4579004) was obtained from the local pharmacy. (22*S*)-budesonide (budesonide epimer A) and (22*R*)-budesonide (budesonide epimer B) were purchased from SimSon Pharma Limited (Mumbai, India). Acetonitrile (of HPLC high grade) was bought from Sigma-Aldrich (Germany). Sodium dihydrogen phosphate product of El-Nasr Pharmaceutical Chemicals (ADWIC), Cairo, Egypt. Orthophosphoric acid was purchased from Prolabo (Paris, France).

### Chromatographic condition

The separation was carried out by a Knauer C_18_ column (150 mm × 4.6 mm, 5 µm particle size). The mobile phase consists of acetonitrile: sodium dihydrogen phosphate (0.04 M) of pH 3.5 adjusted by 0.2 M orthophosphoric acid, in the ratio of (40:60, v/v). The filtration was performed with a 0.45 μm millipore membrane filter and then degassed by sonication for 10 min before injection. The separation was applied at room temperature using a flow rate of 2 mL/min at wavelength 230 nm.

### Procedures

#### Preparation of standard solutions

Standard stock solutions of (100 μg mL^−1^) were created for both BUD and AZL for the utilization in the spectrophotometric methods by dissolving 10 mg of pure drugs into two different 100 mL measuring flasks, and the volume was brought to the mark with acetonitrile. Additional dilutions were carried out for the HPLC procedure to produce the working solutions of (50 μg mL^−1^ for each drug). Furthermore, Standard stock solutions of BUD epimer A and epimer B were prepared by dissolving 10 mg individually into two separate 100 mL volumetric flasks, the volume was completed to the mark with the same solvent. When maintained in the refrigerator at 4 °C, the standard solutions were confirmed to be stable for at least a week.

#### General procedures and construction of calibration graphs

##### HPLC method

Increasing volumes of standard solutions of BUD and AZL were successfully transferred into a series of 10 mL volumetric flasks, yielding solutions with concentrations ranging from (0.4–40.0 μg mL^−1^) for BUD and (0.05–40.0 μg mL^−1^) for AZL. Moreover, standard solutions of both BUD epimers (Epimer A and B) and AZL were transferred into another series of 10 mL volumetric flasks yielding final solutions with the same concentrations range. Prior to injection, the flasks were filled to the mark with the mobile phase, and 20 μL were injected in triplicate at room temperature (25 °C). The calibration curves were constructed by plotting the peak areas against the final concentration of the studied drugs, and the regression equations were created.

##### Spectrophotometric methods

In order to obtain solutions with concentrations range of 6.0–20.0 and 1.0–35.0 μg mL^−1^ for BUD and AZL, respectively, an increasing volume of the standard solution were transferred to a series of 10 mL measuring flasks, which were then diluted to 10.0 mL with acetonitrile. The zero-order absorption spectra for both drugs were recorded against acetonitrile a blank.

For determination of AZL by zero-order direct spectrophotometric method. The absorbance values of AZL were recorded at 290 nm where; there are no overlapped spectra (no absorbance gathered from BUD).

The first-order derivative values were recorded at 265 nm in order to determine BUD via first-derivative spectrophotometry. The calibration graph was created by plotting the derivative amplitudes against the drug concentrations, and the associated regression equation was then created.

Moreover, the first derivative of the ratio spectra was recorded (the spectra of BUD divided by the spectrum of 10 µg mL^−1^ AZL solution) was recorded. The signal at 270 nm for BUD was measured. The calculated values were then plotted against the final concentrations to generate the calibration graph, from which the relevant regression equation was constructed.

#### Determination of the studied drugs in their synthetic laboratory-prepared mixture

To create various laboratory-prepared mixtures of BUD and AZL, accurately measured aliquots of the standard solutions of both drugs were transferred into a set of 10 mL volumetric flasks. Different concentrations of both drugs were utilized to get a constant ratio of (1:4.28) for (BUD: AZL), similar to those employed in pharmaceutical formulation. The volume was completed with the mobile phase for the HPLC method and with acetonitrile for spectrophotometric methods, mixed well, and analyzed as indicated under “Construction of calibration graphs” for each method. Finally, the regression equations and their parameters were then calculated and derived statistically.

#### Application of the proposed methods to the analysis of the studied drugs in their laboratory-prepared dosage form

Into a 25 mL volumetric flask, 10 sprays of rhino aqua (each spray contained 32 µg BUD) and 1.37 mL of Zalastin nasal spray (each mL contained 1000 µg AZL) were transferred, mixed with 15 mL acetonitrile, and sonicated for 10 min. The solution was topped up with acetonitrile, well mixed, filtered, and then proceeded as stated under “Construction of calibration graphs” for each approach.

## Results and discussion

**Fig. 2:**
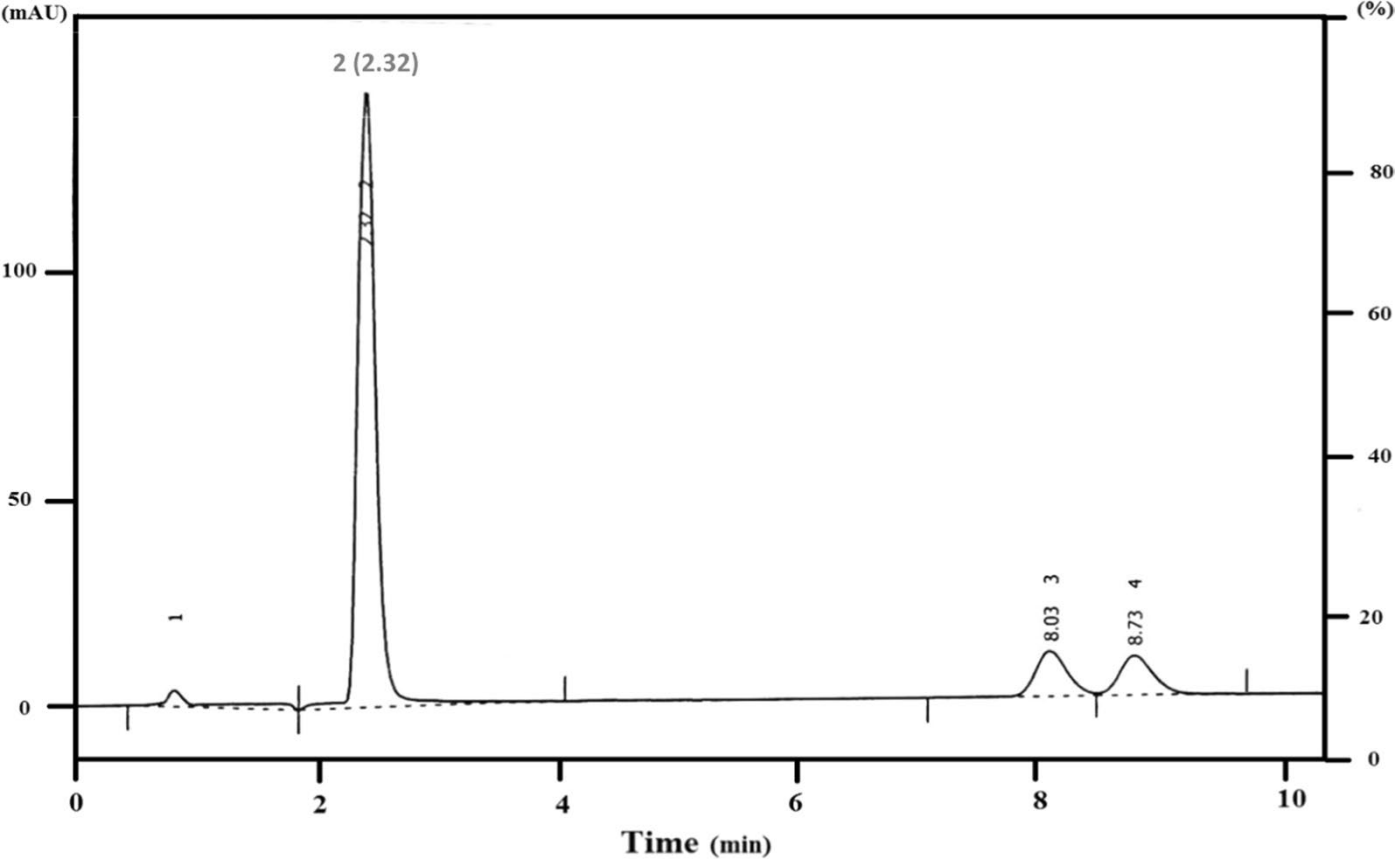
Typical chromatogram for the separation of BUD (30 µg mL^−1^) [BUD Epimer B; 8.03 min, BUD epimer A; 8.73 min) and AZL (30 µg mL^−1^, 2.32 min)]. Where; (1) solvent front, (2) AZL, (3) BUD epimer B and (4) BUD epimer A

A validated chromatographic method was developed for the simultaneous estimation of BUD and AZL. The proposed method provided a highly sensitive and rapid determination of both drugs in their laboratory synthetic mixture and pharmaceutical preparation, as it provided a successful separation with satisfactory resolution (Rs = 13.16, 1.17) between BUD, AZL, and between two epimers of BUD, respectively; within a short elution time (less than 9 min, Rt_BUD epimers(B, A)_ = 8.03, 8.73 min, Rt_AZL_ = 2.32 min) under the optimum selected chromatographic condition as represented in Fig. 2.

**Fig. 3:**
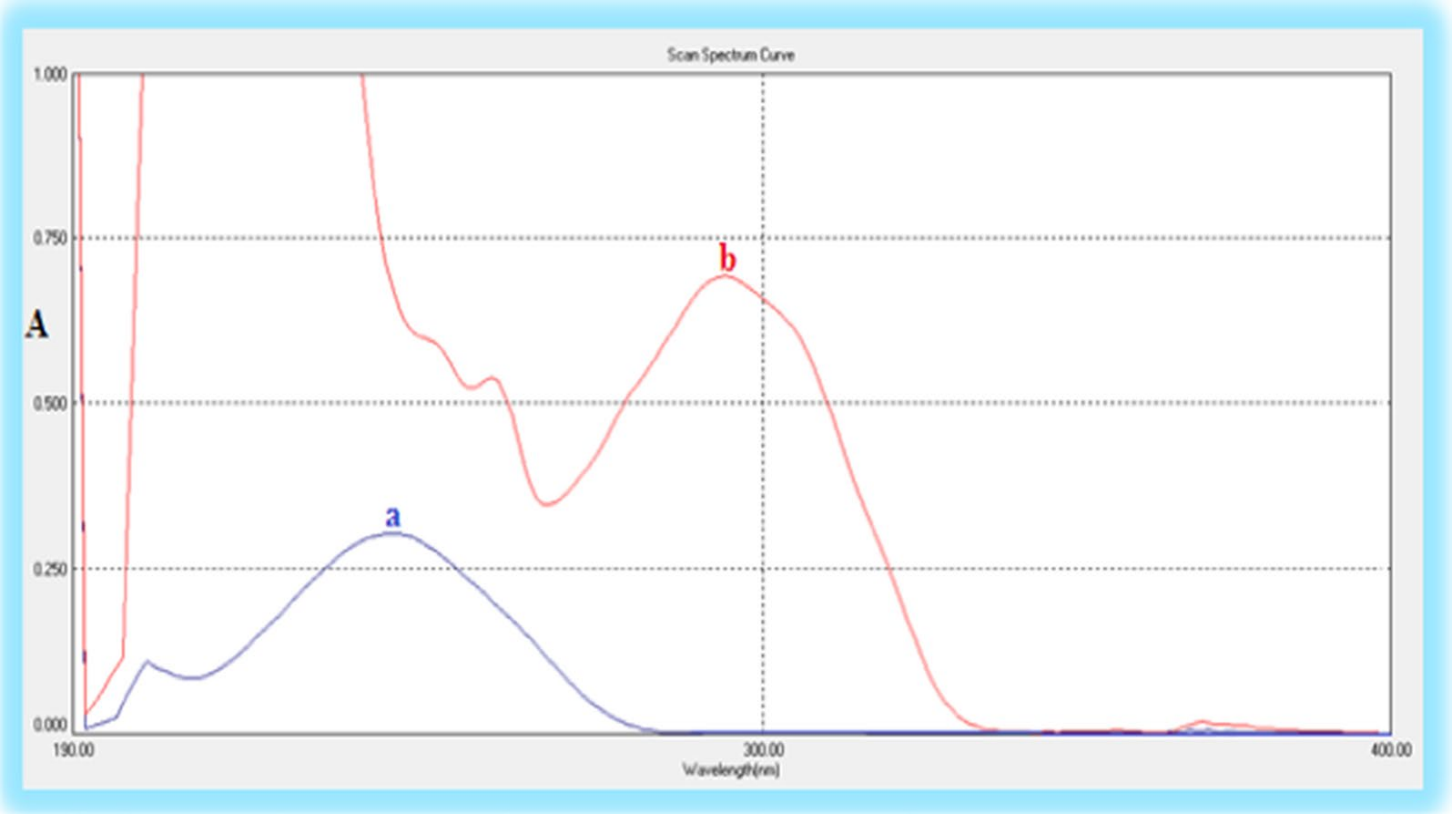
Absorption spectra of **a** BUD (6 μg mL^−1^) and **b** AZL (25.0 μg mL^−1^) in acetonitrile

The UV absorption spectrum of BUD exhibited maxima at 244 nm and AZL possesses UV absorption maxima at 228 nm, 257 nm, and 290 nm as shown in Fig. 3. Thus, conventional UV spectrophotometry could be used for the determination of AZL at the wavelength of 290 nm in presence of BUD, as there is no absorbance observed from BUD at this wavelength. On the other hand, BUD couldn't be measured at the wavelength of 244 nm due to overlapped spectra, so the first derivative and ratio first derivative methods were suggested for simultaneous estimation of both drugs without any interference from each other.

**Fig. 4:**
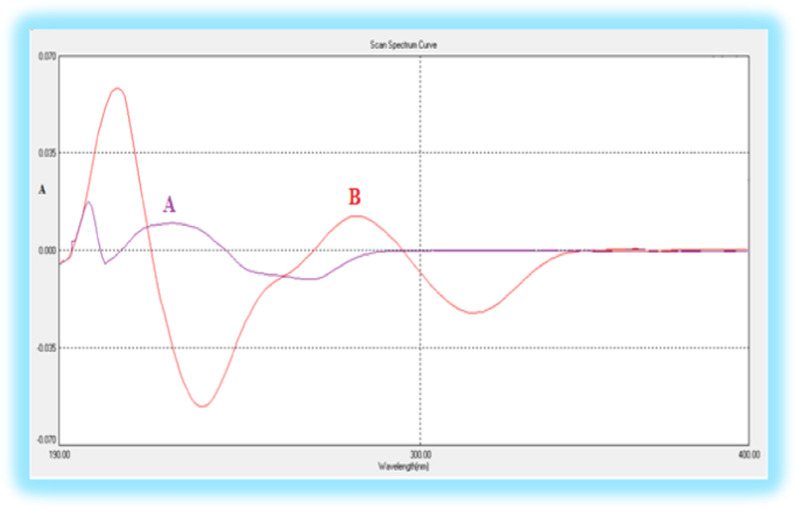
First-order derivative spectra of **a** 6 µg mL^−1^ BUD and **b** 25 µg mL^−1^ AZL in acetonitrile, where BUD measured at 265 nm (zero crossing for AZL)

The first derivative spectra of both BUD and AZL exhibit aspects that may allow the successful determination of BUD in the presence of AZL as shown in Fig. 4. the first derivative intensity at 265 nm (zero-crossing of AZL) was chosen for the simultaneous estimation of BUD in the presence of AZL in the binary mixture.

**Fig. 5:**
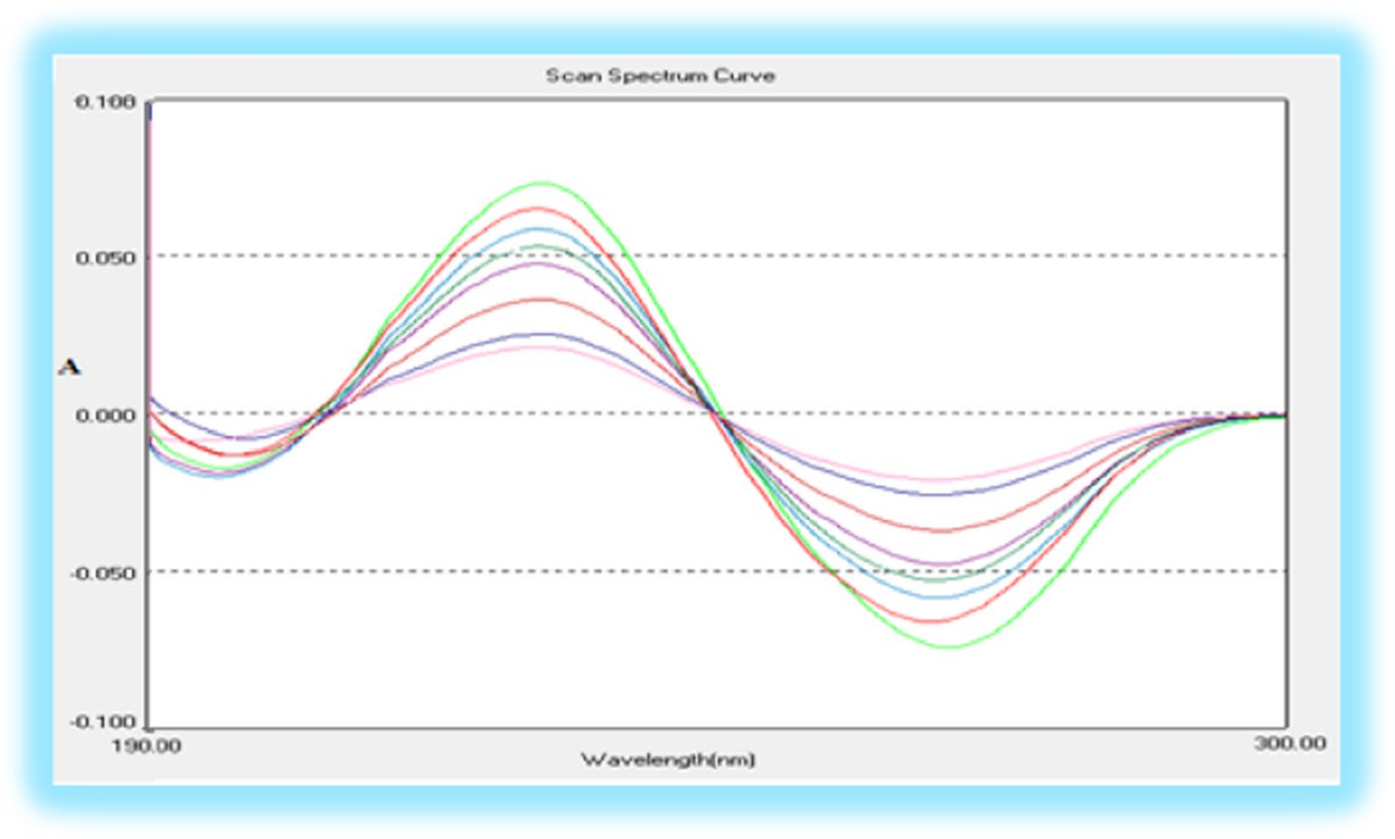
First derivative of the ratio spectra of BUD (6, 8, 10, 12, 14, 16, 18 and 20 µg mL^−1^) when 10.0 µg mL^−1^ AZL was used as divisor (at 233, 270 nm)

In the ratio first derivative spectrophotometry, the first derivative of the ratio spectra of various BUD concentrations (spectra divided by the spectrum of a solution containing 10.0 µg mL^−1^ of AZL) was shown in Fig. 5. Where, the intensity at 233 or 270 nm (^1^DD_233, 270_) in the ratio derivative spectra represent BUD that exists in the solution, so it can be used for its estimation.

As it directly affects the spectral shape, bandwidth, and signal value, the optimal Δλ value is a critical determinant while conducting the first derivative of the ratio spectra, as a result, several values were investigated. It was found that Δλ = 7 nm gave the best shape, the maximum absorbance values, and the best-separated peaks so, it was selected to determine BUD in presence of AZL. Various concentrations were investigated, and various calibration curves were produced, with the purpose of identifying the standard solution as a divisor. Spectra of 10.0 µg mL^−1^ AZL was used as a divisor for the determination of BUD, the best results in terms of sensitivity, signal-to-noise ratio, and repeatability were achieved.

### Optimization of the chromatographic condition

The performance of the utilized chromatographic system of the investigated combination was affected by several parameters, which were evaluated in order to determine the optimum conditions that achieve an efficient separation with satisfactory results.

#### UV-detection

The UV absorption spectra of the investigated drugs exhibited maximum absorption at 244 nm for BUD and 228, 290 nm for AZL (Fig. 3). During the laboratory investigations, several wavelengths were evaluated at 210, 220, 230, 240, 250, and 254 nm. The wavelength at 230 nm was chosen as it shows the highest sensitivity for both drugs.

#### Mobile phase composition

Different alterations in the composition mobile phase were considered so as to change the selectivity of the chromatographic system to provide efficient separation of not only BUD and AZL but also the two epimers of BUD; these modifications include:

##### Type of organic modifier

Several organic modifiers including (methanol and acetonitrile) were investigated to choose the best for the separation. Acetonitrile was chosen, as it allowed separation in shorter elution time with symmetrically good-resolved peaks. On the other side, methanol resulted in band broadening and retardation for both BUD and AZL.

##### The ratio of organic modifier

The influence of different ratios of acetonitrile on the separation efficiency of eluted drugs was tested in the range of (30–50%). It was found that changing the ratio of organic modifier has a great influence on separation on both drugs, and on the two epimers of BUD, as above 42% of acetonitrile, poor separation of both epimers of BUD was gained, as their resolution was less than 1. Below 40% of acetonitrile, both epimers of BUD were retained to unacceptable retention time with lower separation efficiency as revealed by N for both BUD and AZL. So, 40% of acetonitrile was used as the optimum ratio with 60% (0.04 M) of sodium dihydrogen phosphate as shown in Table 1.

**Table 1:** Optimization of chromatographic performance for simultaneous separation of BUD epimers and AZL by HPLC technique

Parameter		N			(D_m_)			(α)		(Rs)	
		BUD_(Epimer B)_	BUD_(Epimer A)_	AZL	BUD_(Epimer B)_	BUD_(Epimer A)_	AZL	BUD_(B)_/AZL	BUD_(B)_/BUD_(A)_	BUD_(B)_/AZL	BUD_(B)_/BUD_(A)_
Ratio of organic modifier A/B*	50:50	2204	2389	449	4.28	4.59	0.59	7.80	1.07	8.98	0.58
	45:55	3702	3598	1010	6.39	6.90	1.31	5.25	1.08	11.60	0.86
	42:58	4133	3995	1038	8.75	9.56	1.68	5.69	1.09	13.37	1.07
	40:60	4460	4202	1032	9.71	10.64	2.09	5.08	1.10	13.16	1.17
	30:70	3734	4101	936	15.97	17.49	3.00	5.83	1.09	14.68	1.13
pH	3.0	4208	3946	995	9.40	10.28	1.68	6.12	1.09	13.81	1.10
	3.5	4460	4202	1032	9.71	10.64	2.09	5.08	1.10	13.16	1.17
	4.0	4383	4154	929	9.34	10.27	2.36	4.35	1.10	11.78	1.20
	5.0	4416	4141	731	8.99	9.83	3.45	2.85	1.09	8.15	1.13
	6.0	4394	4119	775	9.35	10.23	5.30	1.93	1.09	5.22	1.12
Molar strength of buffer	0.02 M	4139	3879	1681	11.53	12.19	4.47	2.73	1.06	11.44	1.38
	0.03 M	4102	3954	1161	11.06	11.99	3.40	3.52	1.08	11.47	1.32
	0.04 M	4460	4202	1032	9.71	10.64	2.09	5.08	1.10	13.16	1.17
	0.05 M	4098	3774	436	8.93	9.88	0.97	10.2	1.11	14.87	0.95

Where: A/B*, A is acetonitrile and B is phosphate buffer (0.04 M)

(N) is number of theoretical plates = 5.45 (t_R_/W_h/2_)^2^

(D_m_) is mass distribution ratio = t_R_ − t_m_/t_m_

(α) is selective factor = D_m2_/D_m1_

(Rs) is resolution = 2Δt_R_/W_1_ + W_2_

t_R_ is the retention time of the substance measured from the point of injection while tm is the retention time of a non-retained marker

W_h/2_ is the peak width at the half height

W_1_ and W_2_ are the width of the peaks of the two components at their bases

##### The pH of the mobile phase

The effect of pH of the mobile phase was investigated across the range (3.0–6.0) by utilizing (0.2 M) orthophosphoric acid. By reviewing the literature it was found that BUD has a log P of 2.73 and a pKa of 13.74 [47], while AZL has a Log P of 3.5 [48] and a pKa of 8.88 [39]. The proposed study revealed that as the pH of the mobile phase was elevated, the AZL peaks appeared unsymmetrical and broader with increased elution time, while BUD was not significantly affected by pH where it was assumed to be fully unionized over the investigated pH range while AZL is ionized and elute very rapid. So, pH 3.5 was selected as the optimum pH for separation, as well-defined symmetrical peaks were eluted with adequate resolution and appropriate elution times as shown in Fig. 2, Table 1.

##### The ionic strength of phosphate buffer

The effect of the different molar concentrations of phosphate buffer on the separation efficiency of eluted drugs was performed in the range from (0.02–0.05 M) at a fixed ratio of (60% phosphate buffer: 40% acetonitrile, v/v), 0.04 M phosphate buffer was chosen as it provided the best separation with the highest efficiency in adequate elution time and appropriate selectivity as shown in Table 1.

#### Flow rate

The flow rate was likewise evaluated from 1.5 to 2.2 mL/min. A flow rate of 2 mL/min was selected as the optimum flow rate afforded efficient separation in an appropriate elution time; below this flow rate, an increased run time was achieved with low sensitivity; above it, the two epimers of BUD were badly resolved from each other, also AZL was co-eluted with solvent front.

The optimum chromatographic condition affected the efficiency of separation of BUD and AZL was achieved by a mobile phase constituted of acetonitrile: sodium dihydrogen phosphate of pH 3.5 adjusted by 0.2 M orthophosphoric acid (40:60, v/v) with flow rate 2 mL/min.

### Analytical performance

The suggested approaches were validated in accordance with ICH Q2 (R1) guidelines [49] concerning the following validation criteria.

#### Linearity and range

The peak area and the drug concentration across the range are correlated linearly throughout the range of (0.4–40.0 µg mL^−1^) and (0.05–40.0 µg mL^−1^) for BUD and AZL, respectively in the HPLC method and between (derivative amplitude & absorbance) and drug concentration throughout the range of (6.0–20.0 µg mL^−1^) and (1.0–35.0 µg mL^−1^) of BUD and AZL, respectively in spectrophotometric methods. Calibrations of both pure epimers (A and B) were performed separately, and their regression equations were derived (Eqs. 1 and 2). Furthermore, the overall BUD concentration was calculated by summation of the AUC of both peaks of the two epimers consequently the data was applied to the linear regression analysis, and Eq. (3) was developed (Table 2).

**Table 2:** Analytical performance data for the simultaneous determination of BUD and AZL by the proposed HPLC and spectrophotometric methods

Drug	BUD					AZL	
Parameter	HPLC method			First derivative	Ratio first derivative	HPLC method	Zero order method
	BUD sum epimer B + A	BUD epimer B	BUD epimer A	265 nm	270 nm		290 nm
Range of linearity (μg mL^−1^)	0.4–40.0			6.0–20.0	6.0–20.0	0.05–40.0	1.0–35.0
Correlation coefficient (*r*)	0.9999						
Slope (*a*)	13.31	13.31	13.31	17 × 10^–4^	38 × 10^–4^	47.48	26 × 10^–3^
Intercept (*b*)	− 0.36	− 0.07	− 0.29	18 × 10^–4^	− 19 × 10^–4^	0.89	34 × 10^–3^
% RSD^a^	0.72	0.75	0.68	0.81	0.85	0.84	0.76
% Error^b^	0.25	0.27	0.24	0.29	0.3	0.30	0.24
S.D. of residuals (S_*y*/*x*_)	0.844	0.429	0.415	19 × 10^–5^	45 × 10^–5^	0.23	26 × 10^–4^
S.D. of intercept (S_*a*_)	0.457	0.233	0.225	22 × 10^–5^	4 × 10^–5^	0.111	136 × 10^–5^
S.D. of slope (S_*b*_)	0.023	0.023	0.022	2 × 10^–5^	5 × 10^–4^	0.006	7 × 10^–5^
LOD (μg mL^−1^)^c^	0.113	0.058	0.056	0.43	0.43	0.008	0.17
LOQ (μg mL^−1^)^d^	0.343	0.175	0.170	1.29	1.32	0.023	0.52

^a^Percentage relative standard deviation

^b^Percentage relative error

^c^Limit of detection

^d^Limit of quantitation

Table 2 displayed analytical performance data for determining BUD and AZL. The following equations were developed by linear regression analysis of the data gathered using the described techniques:

1 $${\text{For}}\;{\text{HPLC}}\;{\text{method:}}\;{\text{PA}} = - 0.07 + 13.31\;{\text{C}}\;\left( {{\text{r}} = 0.9999} \right)\quad {\text{For}}\;{\text{BUD}}\;{\text{epimer}}\;{\text{B}}\;{\text{only,}}$$

2 $${\text{For}}\;{\text{HPLC}}\;{\text{method:}}\;{\text{PA}} = - 0.29 + 13.31\;{\text{C}}\;\left( {{\text{r}} = 0.9999} \right)\quad {\text{For}}\;{\text{BUD}}\;{\text{epimer}}\;{\text{A}}\;{\text{only,}}$$

3 $${\text{For}}\;{\text{HPLC}}\;{\text{method:}}\;{\text{PA}} = - 0.36 + 13.31\;{\text{C}}\;\left( {{\text{r}} = 0.9999} \right)\quad {\text{For}}\;{\text{BUD}}\;\left( {{\text{epimer}}\;{\text{A}} + {\text{B}}} \right),$$

4 $${\text{For}}\;{\text{HPLC}}\;{\text{method:}}\;{\text{PA}} = 0.89 + 47.48\;{\text{C}}\;\left( {{\text{r}} = 0.9999} \right)\quad {\text{For}}\;{\text{AZL}}{.}$$

- For the first derivative: ${\text{DA}} = 18 \times 10^{ - 4} + 17 \times 10^{ - 4} \;{\text{C}}\;\left( {{\text{r}} = 0.9999} \right)\quad {\text{For}}\;{\text{BUD}}\;{\text{at}}\;265\;{\text{nm}}\left( {^{1} {\text{D}}_{265} } \right),$
- For the ratio first derivative: ${\text{DA}} = - 19 \times 10^{ - 4} + 38 \times 10^{ - 4} \;{\text{C}}\;\left( {{\text{r}} = 0.9999} \right)\quad {\text{For}}\;{\text{BUD}}\;{\text{at}}\;270\;{\text{nm}}\;\left( {^{1} {\text{DD}}_{270} } \right),$
- For the zero order: ${\text{A}} = 34 \times 10^{ - 3} + 26 \times 10^{ - 3} \;{\text{C}}\;\left( {{\text{r}} = 0.9999} \right)\quad {\text{For}}\;{\text{AZL}}\;{\text{at}}\;290\;{\text{nm}}{.}$
  where PA is the peak area, C is the concentration of the drug (µg mL^−1^), DA is the derivative amplitude, A is the absorbance and r is the correlation coefficient.

According to the analysis of the data [50], the regression equation's correlation coefficient (r) had a high value, and intercepts (a), slopes (b), standard deviations of intercept (S_a_), standard deviations of slope (S_b_), standard deviations of residuals (Sy/x), percentage relative standard deviation (% RSD), and percentage relative error (% Error) had a small values (Table 2). Low point scattering was observed in the data around the calibration curve.

#### Sensitivity of the method

According to ICH Q2 (R1) guidelines [49], the limit of quantitation (LOQ) and the limit of detection (LOD) were determined by employing the provided equations:

$${\text{LOQ}} = 10\;S_{a} /b\;{\text{and}}\;{\text{LOD}} = 3.3\;S_{a} /b.$$

where *S*_*a*_ = standard deviation of the intercept and *b* = slope of the calibration curve.

In the suggested methods, the values of LOQ and LOD for both BUD epimers B, A, and AZL were calculated and summarized in Table 2.

#### Accuracy

A comparison of the analytical data from the BUD and AZL laboratory assays with those obtained from HPLC comparison techniques was carried out in order to confirm the accuracy of the HPLC method [13, 40]. For statistical data analysis, student’s *t*-test and variance ratio F-test were employed [50], ensuring that there is no significant difference in the findings as shown in Table 3. As well a standard addition method was applied to ensure the validity of the proposed spectrophotometric methods for the simultaneous determination of BUD and AZL in their laboratory-prepared dosage form. The recovery of both drugs (BUD and AZL) separately and in their combination was determined. In Tables 2 and 3; the comparison between the proposed methods and the reported one was performed by adopting Eq. 3 [the summation of the area under the curve (AUC) for both epimers].

**Table 3:** Comparative analytical resulted data for determination of BUD and AZL in pure form by the proposed HPLC and comparison methods

Parameter	BUD		AZL	
	Proposed method	Comparison method [13]	Proposed method	Comparison method [40]
$\overline{\text{X}}$ ± SD	100.29 ± 0.75	99.95 ± 1.36	99.85 ± 0.84	99.61 ± 0.98
*t-*value	0.58 (1.81)		0.45 (1.81)	
F-value	3.28 (4.35)		1.37 (4.35)	

Each result is the mean recovery of three separate determinations

Figures between brackets are the tabulated *t* and F-values at (P = 0.05)

For BUD, the recovery of BUD was calculated by adding a known concentration of the pure drug (7.48 μg mL^−1^) to a previously analyzed nasal spray solution at three different concentrations (6.4, 7.0, and 8.18 μg mL^−1^). For each of the aforementioned nasal spray concentrations, the mentioned concentration of the pure BUD was added in separate flasks and the solution was then reanalyzed to determine the total amount of the drug. The approach was performed in triplicate adopting the procedure as described below “Construction of calibration curves”.

For AZL, the recovery was determined by adding a known amount of pure drug (32.0 μg mL^−1^) to a previously analyzed nasal spray solution at three different concentrations (27.4, 30.0, 35.0 μg mL^−1^) in separate flasks, and the solution was reanalyzed for the total drug concentration. The approach was performed in triplicate adopting the procedure as outlined below “Construction of calibration curves”.

For estimation of BUD and AZL in their combination, the addition of both pure drugs in a ratio of 1:4.28 (7.48 µg mL^−1^ and 32.0 µg mL^−1^) for BUD and AZL, respectively was performed, then previously analyzed nasal spray solutions of both drugs were added at three different concentrations (6.4, 7.0, 8.18), (27.4, 30.0, 35.0) for BUD and AZL, respectively. For each of the aforementioned nasal spray concentrations, the mentioned concentration of both pure drugs was added in separate flasks and the solution was then reanalyzed to determine the total concentration of the drugs. The approach was performed in triplicate utilizing the procedure as mentioned below “Construction of calibration curves”. The obtained results were shown in Additional file 1: Table S1.

#### Precision

The intra-day and inter-day precision for the proposed methods were performed by assaying three different concentrations for both drugs in 1 day and for 5 consecutive days. Where, in the HPLC method analysis of BUD and AZL was performed using three distinct concentrations of (12, 15, and 18 µg mL^−1^) for both BUD and AZL. While in the spectrophotometric methods triplicate assay of BUD and AZL at (10, 13, and 16 µg mL^−1^) and at (12, 15, and 18 µg mL^−1^), respectively. The low values of SD and % RSD as shown in Additional file 1: Table S2 demonstrate the good precision of the suggested approaches.

### Robustness

The suggested chromatographic method’s robustness was assessed to guarantee its reliability in the analytical application. Slight variations in one of the variables were performed while the others were kept constant, including the pH of the mobile phase (3.5 ± 0.2) and the percentage of organic modifiers (40.0% ± 1%). The efficiency of separation and resolution of BUD epimers and AZL was undisturbed by the results gathered from these slight alterations, indicating the reliability of the suggested technique.

#### Applications

##### Analysis of BUD/AZL in their synthetic mixtures and dosage forms

The suggested approaches were furthermore operated for simultaneous estimation of BUD and AZL in the ratio of (1:4.28) for (BUD: AZL) in their laboratory synthetic mixture and their dosage form. The analyses were carried out in triplicate at three different concentrations 1.7, 3.2, and 6.4 μg mL^−1^ for BUD and 6.85, 13.7 and 27.4 μg mL^−1^ for AZL in the HPLC method. As well as 6.4, 7.0, and 8.18 μg mL^−1^ for BUD and 27.4, 30.0, and 35.0 μg mL^−1^ for AZL in spectrophotometric methods; the same procedures were performed as described below “Construction of calibration graphs”. The application of the proposed method for the determination of BUD concentration in the laboratory-prepared mixture and dosage form was afforded by summation of the AUC of the two epimers peaks consequently Eq. (3) was utilized. The obtained statistical data were adequate, as indicated by the low values of SD as summarized in Table 4. The representative chromatograms constructed from the application of both laboratory synthetic mixture and laboratory-prepared dosage form were shown in Additional file 1: Figs. S1 and S2.

**Table 4:** Comparative analytical resulted data from simultaneous determination of BUD and AZL in their laboratory synthetic mixture and laboratory prepared dosage form by the proposed HPLC and spectrophotometry methods

HPLC method								
	Parameter	Laboratory synthetic mixture			Laboratory prepared dosage form			
		Proposed method		Comparison method [13, 40]		Proposed method		Comparison method [13, 40]
BUD + AZL	$\overline{\text{X}}$ ± SD	BUD	99.52 ± 0.21	99.3 ± 0.58	Rhino aqua and Zalastin	BUD	99.39 ± 0.24	100.30 ± 0.87
		AZL	99.85 ± 0.23	99.59 ± 0.61		AZL	100.10 ± 0.67	99.97 ± 0.42
	*t-*value	BUD	0.62 (2.13)			BUD	1.75 (2.13)	
		AZL	0.7 (2.13)			AZL	0.29 (2.13)	
	F-value	BUD	7. 16 (19.0)			BUD	12.61 (19.0)	
		AZL	6.9 (19.0)			AZL	2.62 (19.0)	

Spectrophotometric methods								
		Laboratory synthetic mixture			Laboratory prepared dosage form			
		BUD		AZL		BUD		AZL
		^1^D_265_	^1^DD_270_	^0^D_290_		^1^D_265_	^1^DD_270_	^0^D_290_
BUD + AZL	$\overline{\text{X}}$ ± SD	99.87 ± 0.37	100.03 ± 0.26	99.88 ± 0.25	Rhino aqua and Zalastin	99.86 ± 0.46	100.12 ± 0.52	99.93 ± 0.19

Each result is the mean recovery of three separate determinations

Figures between brackets are the tabulated *t* and F-values at (P = 0.05)

#### Greenness assessment applying AGREE approach

**Fig. 6:**
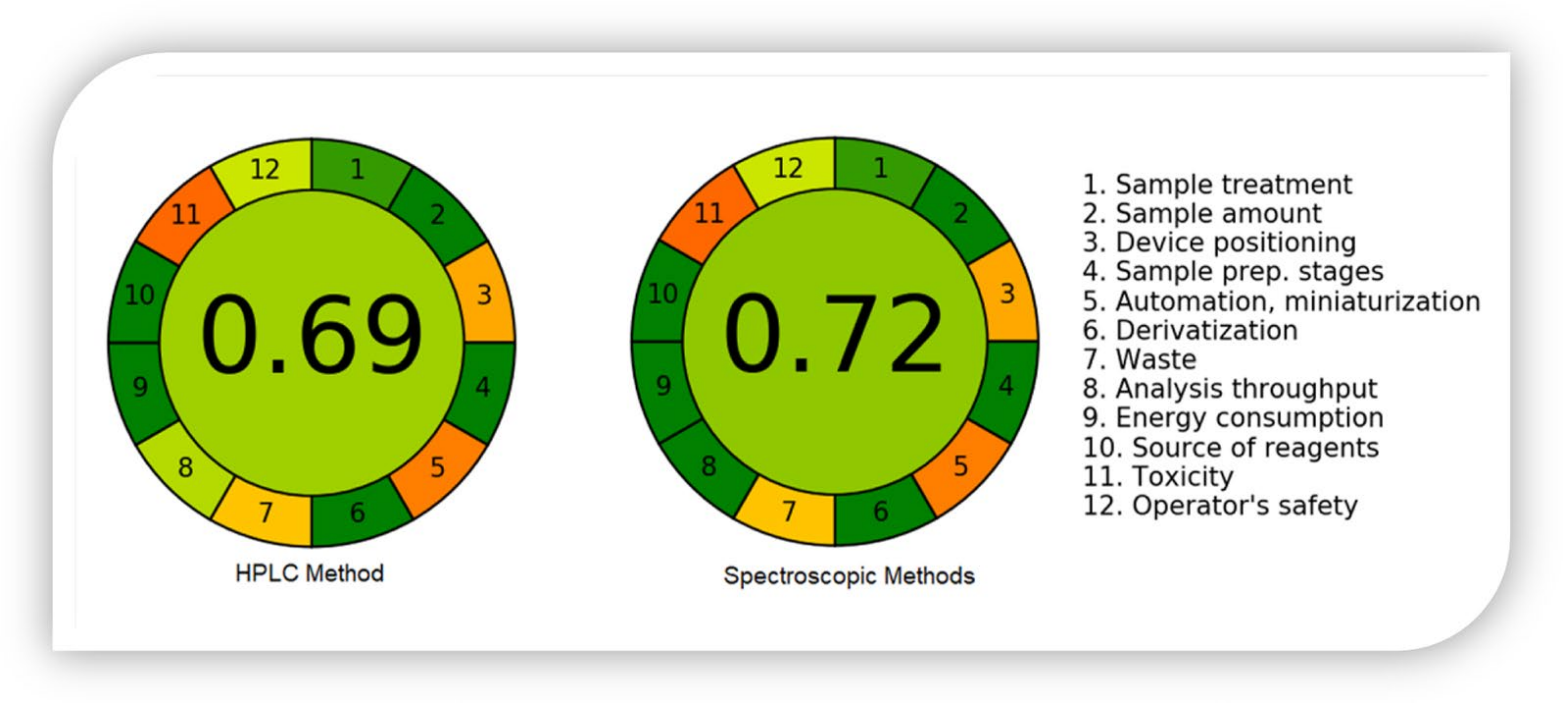
Representative pictograms for AGREE scores for HPLC and spectroscopic methods that obtained using the Analytical Greenness Calculator (AGREE) approach

Various approaches are utilized for evaluating the greenness of analytical assays, however, only AGREE software utilizes all 12 GAC principles for greenness determination [51]. Therefore, the greenness of the approaches was assessed by AGREE: The Analytical Greenness Calculator (version 0.5, Gdansk University of Technology, Gdansk, Poland, 2020). Figure 6 illustrates a representative pictogram for the AGREE score of the proposed chromatographic and spectroscopic methods. For the chromatographic, the AGREE score was calculated to be 0.66, while for the spectroscopic methods utilized for the determination, the AGREE score was found to be 0.72. Both values revealed a good greenness of the proposed approaches.

#### Greenness assessment applying the green analytical procedure index (GAPI) approach

**Fig. 7:**
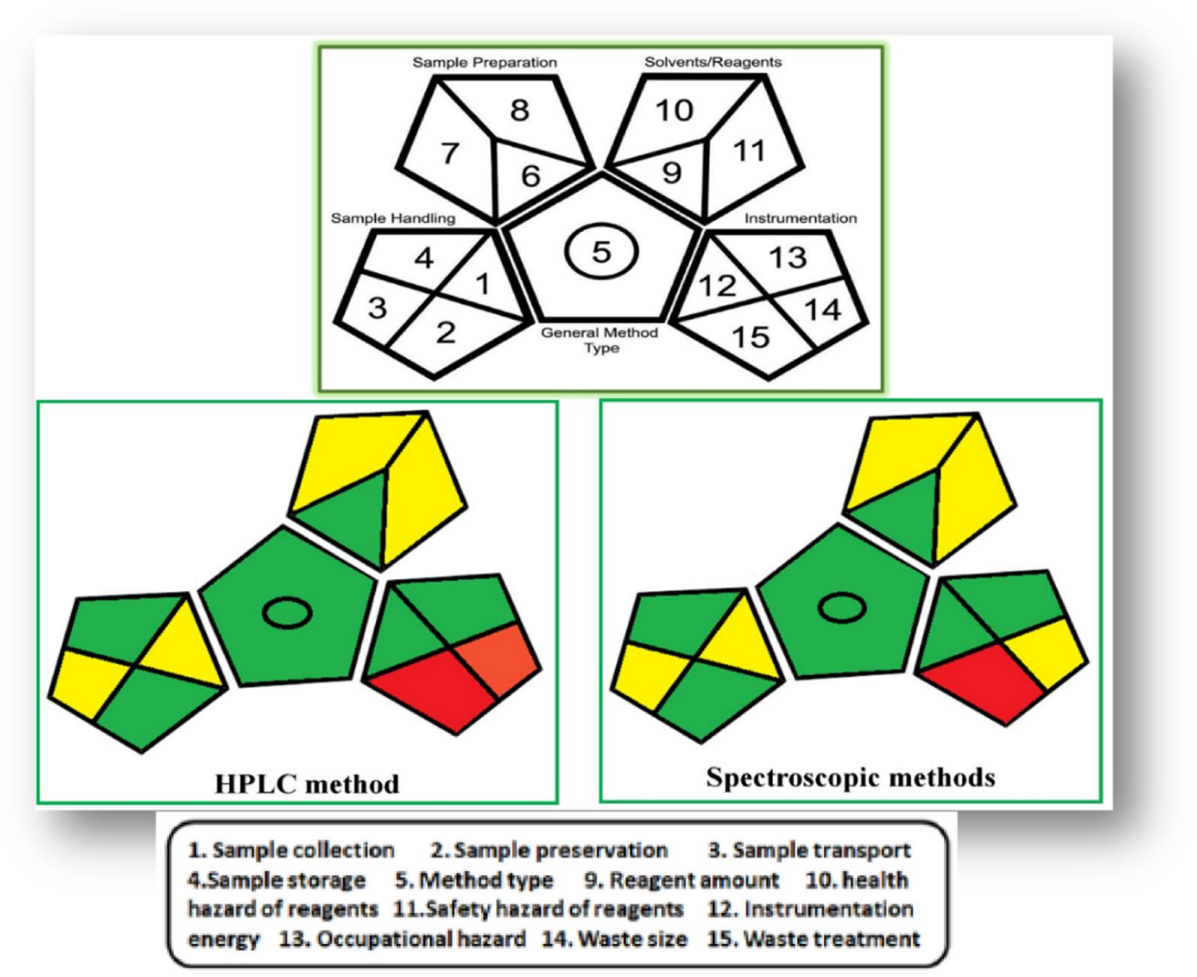
Representative pictograms for HPLC and spectroscopic methods that obtained using green analytical procedure index (GAPI) approach

GAPI is another approach applied to evaluate the greenness of the analytical methodology [52]. Five pentagrams were constructed and colored according to the degree of the environmental effect regarding the different factors. The green color indicates low environmental impact, the yellow color reflects medium environmental impact, and the red color expresses high environmental effect. The excellence of this approach, as well as its capacity to clearly identify the weakest aspects of the conducted operation, motivated us to use it to assess the greenness of the suggested methodologies as shown in (Fig. 7). Both studies revealed that we have to reduce the amount of acetonitrile as a hazardous solvent to enhance the greenness of the proposed methods and decrease waste and toxicity in future studies.

## Conclusion

Efficient, economic, and green chromatographic and spectrophotometric methods were established for the estimation of a novel combination of BUD/AZL that is used successfully for the curing of allergic rhinitis and relieving its symptoms. BUD is an important inhalation corticosteroid for chronic asthmatic patients, and it was found to have an essential role in the inhibition of COVID-19 replication and cytokine production. The proposed spectroscopic and chromatographic methods are applied effectively to estimate and separate this mixture. The separation of BUD epimers and AZL was afforded by the proposed chromatographic approach with excellent performance in a relatively short elution time and satisfactory resolution. Besides, three spectrophotometric methods were utilized for the estimation of this mixture by zero order, first order derivative, and ratio first derivative methods. The suggested methods were validated and further applied for the quantification of BUD/AZL in their laboratory-prepared mixture and dosage form according to the recommended ratio of 1:4.28. The AGREE and GAPI metrics were approved an excellent greenness of the proposed approaches.

## Supplementary Information

**Additional file 1: Figure S1.** Typical Chromatogram for the separation of BUD (6.4 µg mL^−1^) [BUD Epimer B; 8.03 min, BUD Epimer A; 8.73 min) and AZL (27.4 µg mL^−1^, 2.32 min) in their laboratory synthetic mixture. Where; (1) Solvent Front, (2) AZL (3) BUD Epimer B (4) BUD Epimer A. **Figure S2.** Typical Chromatogram for the separation of BUD (6.4 µg mL^−1^) [BUD Epimer B; 8.03 min, BUD Epimer A; 8.8 min) and AZL (27.4 µg mL^−1^, 2.32 min in laboratory prepared dosage form (Rhino Aqua and Zalastin). Where; (1) Solvent Front, (3) AZL (5) BUD Epimer B (6) BUD Epimer A. **Table S1.** Standard Addition Test Results for Spectrophotometric First Derivative, Ratio First Derivative and Zero Order for Determination of BUD and AZL in their Nasal Spray Solutions. **Table S2.** Intra-day and inter-day precision data for the determination of BUD and AZL in pure form by the HPLC and methods.

## Data Availability

Datasets generated and/or analyzed during the current study are available from the corresponding author upon reasonable request.

## Abbreviations

A: Absorbance
ACE2: Angiotensin-converting enzyme 2
AUC: The area under the curve
AZL: Azelastine
BUD: Budesonide
COPD: Chronic obstructive pulmonary disease
C: Concentration
r: Correlation coefficient.
COVID-19: Coronavirus disease 2019
DA: Derivative amplitude
EKCC: Electrokinetic capillary chromatography
GC: Gas chromatography
GAPI: Greenness assessment applying the green analytical procedure index
HPLC: High-performance liquid chromatography
HPTLC: High-performance thin-layer chromatography
HCoV-229E: Human coronavirus 229E
ICS: Inhaled corticosteroids
IFN: Interferon
a: Intercepts of the calibration curve.
LC/MS/MS: Liquid chromatography with tandem mass spectrometry
LOQ: Limit of quantitation
LOD: Limit of detection
µg mL^−^^1^: Microgram per millimeter
PA: Peak area
% RSD: Percentage relative standard deviation
% Error: Percentage relative error
b: Slopes of the calibration curve
S_a_: Standard deviation of the intercept
S_b_: Standard deviation of the slope
Sy/x: Standard deviations of residuals
AGREE: The Analytical Greenness Calculator
EKB: The Egyptian Knowledge Bank
STDF: The Science, Technology & Innovation Funding Authority
TNSS: Total nasal symptom score
UPLC/MS/MS: Ultra-high-performance liquid chromatography with tandem mass spectrometry
