# Supplementary material for: Insights of different analytical approaches for estimation of budesonide as COVID-19 replication inhibitor in its novel combinations: green assessment with AGREE and GAPI approaches
Source: BMC Chem. 2023 Mar 14;17(1):17. doi: 10.1186/s13065-023-00936-z (PMC10013279; doi:10.1186/s13065-023-00936-z)
Supplement: Supplementary file 1 — Additional file 1: Figure S1. Typical Chromatogram for the separation of BUD (6.4 µg mL−1) [BUD Epimer B; 8.03 min, BUD Epimer A; 8.73 min) and AZL (27.4 µg mL−1, 2.32 min) in their laboratory synthetic mixture. Where; (1) Solvent Front, (2) AZL (3) BUD Epimer B (4) BUD Epimer A. Figure S2. Typical Chromatogram for the separation of BUD (6.4 µg mL−1) [BUD Epimer B; 8.03 min, BUD Epimer A; 8.8 min) and AZL (27.4 µg mL−1, 2.32 min in laboratory prepared dosage form (Rhino Aqua and Zalastin). Where; (1) Solvent Front, (3) AZL (5) BUD Epimer B (6) BUD Epimer A. Table S1. Standard Addition Test Results for Spectrophotometric First Derivative, Ratio First Derivative and Zero Order for Determination of BUD and AZL in their Nasal Spray Solutions. Table S2. Intra-day and inter-day precision data for the determination of BUD and AZL in pure form by the HPLC and methods. [file 13065_2023_936_MOESM1_ESM.docx]

**Figures**

**
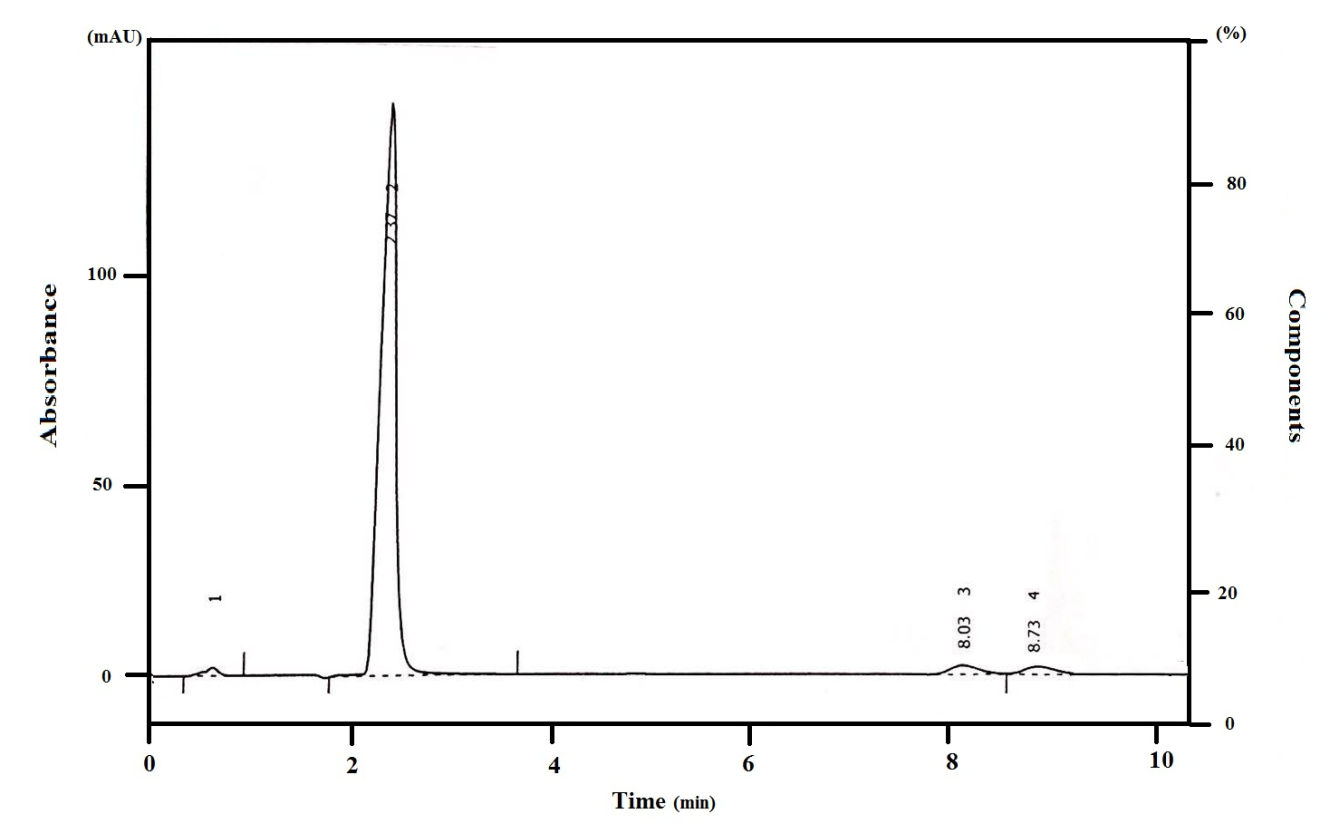
**

**2 (2.32)**

**Fig. S1. Typical Chromatogram for the separation of BUD (6.4 µg.mL^-1^) [BUD Epimer B; 8.03 min, BUD Epimer A; 8.73 min) and AZL (27.4 µg. mL^-1^, 2.32 min) in their laboratory synthetic mixture. Where; (1) Solvent Front, (2) AZL (3) BUD Epimer B (4) BUD Epimer A.**


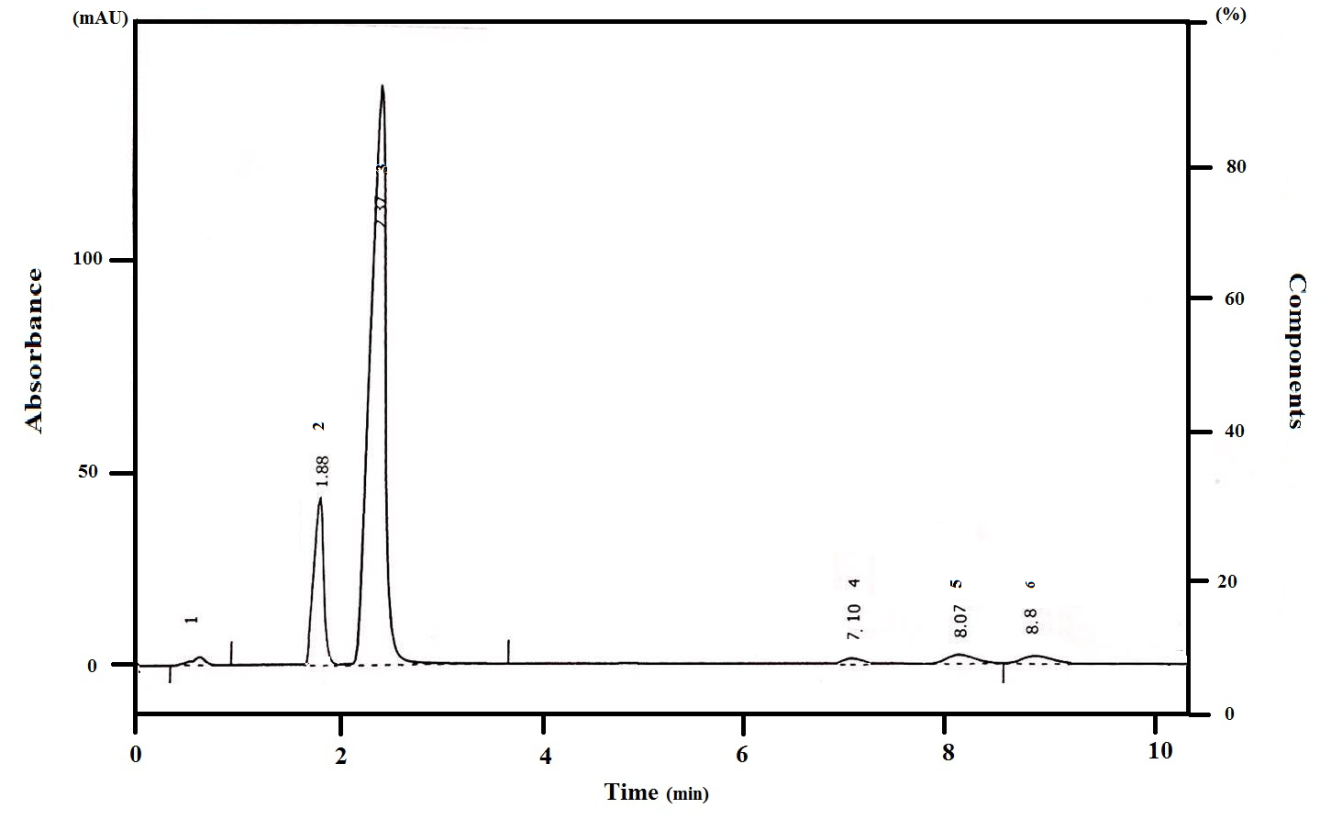


**2 (2.32)**

**Fig. S2. Typical Chromatogram for the separation of BUD (6.4 µg.mL^-1^) [BUD Epimer B; 8.03 min, BUD Epimer A; 8.8 min) and AZL (27.4 µg. mL^-1^, 2.32 min** **in laboratory prepared dosage form (Rhino Aqua and Zalastin). Where; (1) Solvent Front, (3) AZL (5) BUD Epimer B (6) BUD Epimer A.**

**Tables**

**Table S1: Standard Addition Test Results for Spectrophotometric First Derivative, Ratio First Derivative and Zero Order for Determination of BUD and AZL in their Nasal Spray Solutions.**

| **Drug** | **Sample concentration** | | | **Concentration of added standard** | **Recovery ± RSD % of added drug** | **Mean recovery ± RSD % of added drug** |
| --- | --- | --- | --- | --- | --- | --- |
|  |  | | |  |  |  |
| **BUD** | ^1^D_265_ | 7.48 | | 6.40 | 99.19 ± 0.89 | 100.02 **±** 0.74 |
|  |  | 7.48 | | 7.00 | 100.61 ± 0.72 |  |
|  |  | 7.48 | | 8.18 | 100.28 ± 0.60 |  |
|  | ^1^DD _270_ | 7.48 | | 6.40 | 99.58 ± 0.74 | 100.07 ± 0.55 |
|  |  | 7.48 | | 7.00 | 100.37 ± 0.57 |  |
|  |  | 7.48 | | 8.18 | 100.25 ± 0.34 |  |
| **AZL** | D^0^ _290_ | | 32.00 | 27.40 | 100.59 ± 0.88 | 99.87 ± 0.59 |
|  |  |  | 32.00 | 30.00 | 98.88 ± 0.71 |  |
|  |  |  | 32.00 | 35.00 | 100.15 ± 0.17 |  |
| **BUD + AZL** | ^1^D_265_ | | 7.48 + 32.00 | 6.40 + 27.40 | 99.64 ± 0.71 | 100.19 **±** 0.83 |
|  |  |  | 7.48 + 32.00 | 7.00 + 30.00 | 100.74 ± 0.94 |  |
|  |  |  | 7.48 + 32.00 | 8.18 + 35.00 | 100.34 ± 0.58 |  |
|  | ^1^DD _270_ | | 7.48 + 32.00 | 6.40 + 27.40 | 99.58 ± 0.74 | 100.2 ± 0.6 |
|  |  |  | 7.48 + 32.00 | 7.00 + 30.00 | 100.64 ± 0.5 |  |
|  |  |  | 7.48 + 32.00 | 8.18 + 35.00 | 100.25 ± 0.34 |  |
|  | D^0^ _290_ | | 7.48 + 32.00 | 6.40 + 27.40 | 100.59 ± 0.88 | 99.87 ± 0.59 |
|  |  |  | 7.48 + 32.00 | 7.00 + 30.00 | 98.88 ± 0.71 |  |
|  |  |  | 7.48 + 32.00 | 8.18 + 35.00 | 100.15 ± 0.17 |  |

Where:

- ^1^D_265_  is the first derivative measured at 265 nm.
- ^1^DD_270_ is the ratio first derivative measured at 270 nm.
- D^0^_290_ is zero order measured at 290 nm.

**Table S2: Intra-day and inter-day precision data for the determination of BUD and AZL in pure form by the HPLC and methods.**

| **HPLC method** | | | | | | | | | | | | | | | |
| --- | --- | --- | --- | --- | --- | --- | --- | --- | --- | --- | --- | --- | --- | --- | --- |
|  | | | | **BUD**  **Concentration (μg. mL^-1^)** | | | | | | **AZL**  **Concentration (μg. mL^-1^)** | | | | | |
|  | | | | **12** | | **15** | | **18** | | **12** | | **15** | | **18** | |
|  |  |  |  |  | |  | |  | |  | |  | |  | |
| **Intra-day** | | **$\bar{\text{X}}$^a^** | | 99.97 | | 99.98 | | 99.92 | | 100.26 | | 99.99 | | 100.04 | |
|  |  | **± SD** | | 0.4 | | 0.38 | | 0.36 | | 0.34 | | 0.21 | | 0.35 | |
|  |  | **% RSD** | | 0.4 | | 0.38 | | 0.36 | | 0.34 | | 0.21 | | 0.35 | |
|  |  | **% Error** | | 0.23 | | 0.22 | | 0.21 | | 0.19 | | 0.12 | | 0.2 | |
| **Inter-day** | | **$\bar{\text{X}}$^a^** | | 99.89 | | 99.99 | | 99.75 | | 100.16 | | 99.85 | | 99.72 | |
|  |  | **± SD** | | 0.55 | | 0.41 | | 0.42 | | 0.53 | | 0.41 | | 0.4 | |
|  |  | **% RSD** | | 0.55 | | 0.41 | | 0.42 | | 0.53 | | 0.41 | | 0.4 | |
|  |  | **% Error** | | 0.32 | | 0.24 | | 0.24 | | 0.14 | | 0.24 | | 0.23 | |
| **BUD Concentration (μg. mL^-1^)** | | | | | | | | | | | | | | | |
|  | | | | **First derivative at 265 nm** | | | | | | **Ratio first derivative at 270 nm** | | | | | |
|  | | | | **10.0** | | **13.0** | | **16.0** | | **10.0** | | **13.0** | | **16.0** | |
| **Intra-day** | | **$\bar{\text{X}}$^a^** | | 100.02 | | 99.96 | | 99.84 | | 100.01 | | 100.26 | | 99.89 | |
|  |  | **± SD** | | 0.39 | | 0.28 | | 0.48 | | 0.29 | | 0.33 | | 0.35 | |
|  |  | **% RSD** | | 0.39 | | 0.28 | | 0.48 | | 0.29 | | 0.33 | | 0.25 | |
|  |  | **% Error** | | 0.23 | | 0.16 | | 0.28 | | 0.17 | | 0.19 | | 0.15 | |
|  |  |  | |  | |  | |  | |  | |  | |  | |
| **Inter-day** | | **$\bar{\text{X}}$^a^** | | 99.84 | | 100.05 | | 99.81 | | 99.74 | | 100.22 | | 99.69 | |
|  |  | **± SD** | | 0.48 | | 0.49 | | 0.49 | | 0.53 | | 0.38 | | 0.46 | |
|  |  | **% RSD** | | 0.48 | | 0.49 | | 0.49 | | 0.53 | | 0.38 | | 0.46 | |
|  |  | **% Error** | | 0.28 | | 0.29 | | 0.28 | | 0.3 | | 0.22 | | 0.27 | |
| **AZL concentration (μg. mL^-1^)**  **zero order measurement at 290 nm** | | | | | | | | | | | | | | | |
|  | | | **12.0** | | **15.0** | | **18.0** |  |  | | **12.0** | | **15.0** | | **18.0** |
| **Intra-day** | **$\bar{\text{X}}$^a^** | | 99.88 | | 100.18 | | 99.86 | **Inter-day** | **$\bar{\text{X}}$^a^** | | 100.12 | | 100.09 | | 99.86 |
|  | **± SD** | | 0.48 | | 0.26 | | 0.19 |  | **± SD** | | 0.78 | | 0.39 | | 0.27 |
|  | **% RSD** | | 0.48 | | 0.26 | | 0.19 |  | **% RSD** | | 0.78 | | 0.39 | | 0.27 |
|  | **% Error** | | 0.28 | | 0.15 | | 0.11 |  | **% Error** | | 0.45 | | 0.23 | | 0.15 |

- ^a^ Each result is the mean recovery of three separate determinations.
